# Synthetic and natural antioxidants attenuate cisplatin-induced vomiting

**DOI:** 10.1186/s40360-016-0110-9

**Published:** 2017-01-13

**Authors:** Javaid Alam, Fazal Subhan, Ihsan Ullah, Muhammad Shahid, Gowhar Ali, Robert D. E. Sewell

**Affiliations:** 1 Department of Pharmacy, University of Peshawar, Peshawar, 25120 Khyber Pakhtunkhwa Pakistan; 2 Department of Pharmacy, University of Swabi, Swabi, Pakistan; 3 Cardiff School of Pharmacy and Pharmaceutical Sciences, Cardiff University, Cardiff, CF103NB UK

**Keywords:** Emesis, Antiemetic, Chemotherapy, *Bacopa monnieri*, Grape seed proanthocyanidin, *N*-(2-mercaptopropionyl) glycine, Vitamin C, Free radical scavenging assay, Correlation

## Abstract

**Background:**

Synthetic and natural antioxidants including *Bacopa monnieri* (L.) Pennell (Scrophulariaceae) which also possess anti-dopaminergic properties, have been proposed to be useful for emetogenic chemotherapy. In this study, synthetic [*N*-(2-mercaptopropionyl) glycine (MPG), vitamin C (Vit-C)] and natural [grape seed proanthocyanidin (GP), *B. monnieri n*-butanolic fraction (BM-ButFr)] antioxidants and their combinations were evaluated against cisplatin-induced emesis in pigeons during a 24 h observation period.

**Methods:**

Emesis was induced using cisplatin (7.0 mg/kg, i.v). MPG (10, 20, 30 mg/kg), Vit-C (100, 200, 300 mg/kg), GP (50, 100, 150 mg/kg) and BM-ButFr (5, 10, 20 mg/kg) and their combinations were administered i.m., 15 min before cisplatin administration. The number of vomiting bouts, retching, emetic latency and % weight loss were recorded to assess antiemetic potential. Antioxidant activity was evaluated by the DPPH free radical scavenging assay (FRSA).

**Results:**

Significant attenuation of vomiting bouts, retching, % weight loss along with an increase in latency was produced by all the antioxidants and their combinations compared to cisplatin alone and this is the first report of this activity of GP in pigeons. Low EC_50_ values in the FRSA for MPG (67.66 μg/mL), Vit-C (69.42 μg/mL), GP (6.498 μg/mL) and BM-ButFr (55.61 μg/mL) compared to BHT standard (98.17 μg/mL) demonstrated their radical scavenging capacity. Correlation between the antioxidant activity and antiemetic efficacy disclosed a high degree of correlation for the tested antioxidants.

**Conclusion:**

The selected synthetic and natural antioxidants and their combinations were able to attenuate cisplatin-induced vomiting, which correlated with their potent in vitro antioxidant activity.

**Electronic supplementary material:**

The online version of this article (doi:10.1186/s40360-016-0110-9) contains supplementary material, which is available to authorized users.

## Background

Nausea and vomiting are the most distressing and commonly occurring side effects of chemotherapeutic agents [1]. Indeed, chemotherapy can induce both acute and delayed phases of nausea and vomiting [2]. In pigeons and piglets for instance, the acute phase lasts for 8–16 h while the delayed phase may endure for 48–58 h [3]. However, in humans, the acute and delayed phases persist for 24 and 7 days, respectively [4]. The mechanisms underlying emesis have been investigated in carnivores such as ferrets, dogs [5], and cats [6], insectivores like *Suncus murinus* (musk shrew) [7] and *Cryptotis parva* (least shrew) (Soricidae) [8, 9]. Similarly, birds, notably pigeons, also display clear-cut emetic responses to copper sulphate, glucagon, digoxin [10], theophylline [11] and amantadine [12].

Cisplatin is an effective chemotherapeutic agent indicated for the management of different malignancies including ovarian [13], head and neck [14], testicular and bladder carcinomas [15]. Its use is associated with many side effects, of which vomiting is distinctly the most distressing [16]. It decreases the plasma levels of various antioxidants [17] and also generates both oxidative and nitrosative stress [18, 19]. The oxidative stress component plays a significant role in cisplatin-induced side effects as well as various other complications [20].

Antioxidants are effective in reducing oxidative stress evoked by cisplatin [21] and they play a pivotal role in protecting against cisplatin elicited nephrotoxicity [22], hepatotoxicity [23] and ototoxicity [24]. A range of antioxidants including vitamin C, *N*-2-mercaptopropionyl glycine (MPG, also named tiopronin), glutathione and vitamin E are known to be effective in cisplatin emetogenesis [25]. In this context, the synthetic antioxidant MPG, reduces cisplatin and pyrogallol provoked vomiting in *Suncus murinus* [26]. Proanthocyanidin (GP) an antioxidant flavonoid [27], not only possesses neuroprotective activity in humans [28] and animals [29], but also initiates a reduction of pica behavior (eating of non-food substances as a model of simulated emesis) in rats [30]. Additionally, it is effective against cisplatin-induced nephrotoxicity and hepatotoxicity [31, 32], provides cardioprotection and enhances cognitive performance [33]. Similarly, *Bacopa monnieri* (L.) Pennell (Scrophulariaceae), a reputed nootropic plant and a rich source of bacosides has strong antioxidant [34] and neuroprotective properties [35].

Although previous studies advocate the effectiveness of antioxidants as antiemetics [25, 36], there is no report available in the literature showing any direct correlation between antiemetic propensity and antioxidant activity in the pigeon model for emetogenesis. The aim of this study therefore was threefold: firstly to determine any antiemetic activity of selected natural and synthetic antioxidants either alone or in combination. Secondly to evaluate their antioxidant potential and thirdly, to establish any possible correlation between their antioxidant and antiemetic activities.

## Methods

### Animals

Pigeons of either sex (mix breed, Department of Pharmacy, University of Peshawar, Pakistan) weighing between 200–400 g were used. They were acclimatized 24 h before the start of the experiment and were maintained at 22–26 °C on a 12 h light-dark cycle. Food and water were provided *ad libitum*. The experiments were performed in accordance with the UK Animals (Scientific Procedures) Act 1986 and were approved by the Ethical Committee of the Department of Pharmacy, University of Peshawar (Reference No. 14/EC-12/Pharm).

### Chemicals and standards

Cisplatin (Korea United Pharm. Inc. Korea), analytical grade methanol (Sigma-Aldrich, Switzerland), 2,2-diphenyl-1-picrylhydrazyl (DPPH; Sigma-Aldrich, Germany), vitamin C (Vit-C; Sigma-Aldrich, Germany), butylated hydroxytoluene (BHT; Sigma-Aldrich, Germany), grape seed proanthocyanidin extracts (GP; Shaanxi Run-time Biotechnology Development Co. Ltd, Xian, China), *N*-(2-mercaptopropionyl) glycine (MPG; Sigma-Aldrich, Germany), metoclopramide (GlaxoSmithKline).

### Preparation of *n*-butanolic fraction of *B. monnieri* extract

Whole plant of *B. monnieri* was collected in November, 2010 from Rumalee stream near Quaid-e-Azam University, Islamabad, Pakistan. It was authenticated by Prof. Dr. Muhammad Ibrar of the Department of Botany, University of Peshawar and a specimen was deposited in the herbarium of the same Department with a voucher No 7421. The aerial parts were separated, shade dried and coarsely powdered. They were extracted with methanol in a Soxhlet apparatus and further fractionated to obtain the *n*-butanolic fraction (BM-ButFr) which is reported to be rich in bacosides [37]. Bacosides are the major active constituents of *B. monnieri* and they are considered to be responsible for its myriad pharmacological properties [38].

### Antiemetic activity

#### Preparation of drug solutions

Cisplatin was dissolved in saline at 65–75 °C with continuous shaking and was cooled before administration. GP, MPG, Vit-C and BM-ButFr were dissolved in sterile normal saline by gentle agitation and sonicated until uniform solutions were obtained. These solutions were then immediately administered by the intramuscular route (i.m.).

#### Induction of emesis

The maximal (100%) emetic dose of cisplatin (7.0 mg/kg) was used as an emetic challenge [39]. It was administered intravenously via the brachial vein and pigeon behavior was recorded for 24 h by video recorder. Any response with or without oral expulsion of gastric contents was considered as one bout (vomiting episode) and if occurring after a gap of 1 min from another bout, they were considered as separate vomiting episodes with 2 up to 80 numbers of retching (emetic behaviors) [40]. The latency to first vomit, the number of bouts, retching and % weight loss were all recorded.

#### Drug treatment

Emesis was induced by administering cisplatin (7.0 mg/kg) intravenously. MPG, Vit-C, GP and BM-ButFr or their combination as well as metoclopramide used as the standard antiemetic agent, were administered as pretreatments 15 min i.m. prior to cisplatin administration. The different doses of the tested compounds were selected according to previous studies [25, 39, 41, 42]. The animals were divided into the following groups:

- Group I: Cisplatin control (7.0 mg/kg), *n* = 8.
- Group II: Metoclopramide (30 mg/kg), *n* = 8.
- Group III: MPG (10, 20, and 30 mg/kg), *n* = 8 each per dose.
- Group IV: Vit-C (100, 200, and 300 mg/kg), *n* = 8 each per dose.
- Group V: GP (50, 100, and 150 mg/kg), *n* = 8 each per dose.
- Group VI: BM-ButFr (5, 10, and 20 mg/kg), *n* = 8 each per dose.
- Group VII: Combination of MPG (10 mg/kg) plus Vit-C (200 mg/kg), *n* = 7.
- Group VIII: Combination of BM-ButFr (10 mg/kg) plus GP (100 mg/kg), *n* = 8.
- Group IX: Combination of GP (100 mg/kg) plus Vit-C (200 mg/kg), *n* = 8.

The percentage reduction in the frequency of cisplatin-induced vomiting bouts was calculated as:

$$ \%\ \mathrm{Reduction} = \left(1\ \hbox{--}\ \mathrm{mean}\ \mathrm{number}\ \mathrm{of}\ \mathrm{bouts}\ \mathrm{after}\ \mathrm{treatment}/\mathrm{mean}\ \mathrm{number}\ \mathrm{of}\ \mathrm{bouts}\ \mathrm{of}\ \mathrm{untreated}\ \mathrm{control}\right) \times 100 $$

#### DPPH (2,2-diphenyl-1-picrylhydrazyl) free radical scavenging activity in vitro

The antioxidant activities of MPG, Vit-C, GP and BM-ButFr were evaluated by the DPPH free radical scavenging assay [43, 44]. Briefly, 2.0 mL of methanolic 0.1 mM DPPH free radical solution was added to 1.0 mL of different concentrations (1.0, 10, 30, 50, 100, 200, 500 μg/mL) of GP, MPG, Vit-C, BM-ButFr or standard (BHT: butylated hydroxytoluene) in methanol. The solutions were shaken thoroughly, incubated in the dark at ambient temperature for 30 min and absorbance was measured at 517 nm using a UV/Visible spectrophotometer (Lambda 25, PerkinElmer, USA). The % scavenging of DPPH free radicals was calculated as follows:

$$ \%\ \mathrm{of}\ \mathrm{DPPH}\ \mathrm{free}\ \mathrm{radical}\ \mathrm{scavenging}\ \mathrm{activity} = \left[\left({\mathrm{A}}_{\mathrm{I}}\hbox{-} {\mathrm{A}}_{\mathrm{I}\mathrm{I}}/{\mathrm{A}}_1\right) \times 100\right] $$

Where A_I_ is the absorbance of the control reaction and A_II_ is the absorbance in the presence of sample.

The EC_50_, defined as the concentration of antioxidant causing 50% loss of DPPH activity was calculated from the graph of absorbance versus respective concentrations using non-linear regression analysis. All experiments were performed in triplicate.

### Statistical analysis

Data were expressed as mean ± S.E.M (*n* = 7–8) and analyzed by one way ANOVA followed by Tukey’s multiple comparison using GraphPad Prism 5 (GraphPad Software Inc. San Diego CA, USA). Correlation analysis of antioxidant activity versus antiemetic activity of each antioxidant was carried out using the Pearson’s correlation and regression program in Minitab version 17.1.0 (Minitab Inc. State College, PA 16801 USA).

## Results

### Antiemetic activity of N-(2-mercaptopropionyl) glycine (MPG), vitamin C (Vit-C), grape seed proanthocyanidin (GP), and B. monnieri n-butanolic fraction (BM-ButFr) as well as MPG + Vit-C, BM-ButFr + GP and GP + Vit-C combinations

**Fig. 1:**
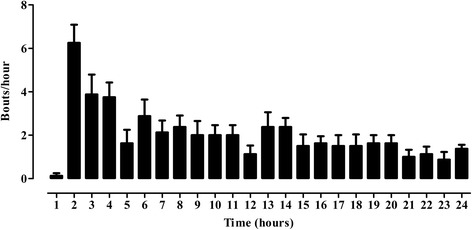
Cisplatin (7.0 mg/kg i.v) induced vomiting bouts in pigeons during a 24 h observation period. Each bar represents mean ± S.E.M (*n* = 8)

**Fig. 2:**
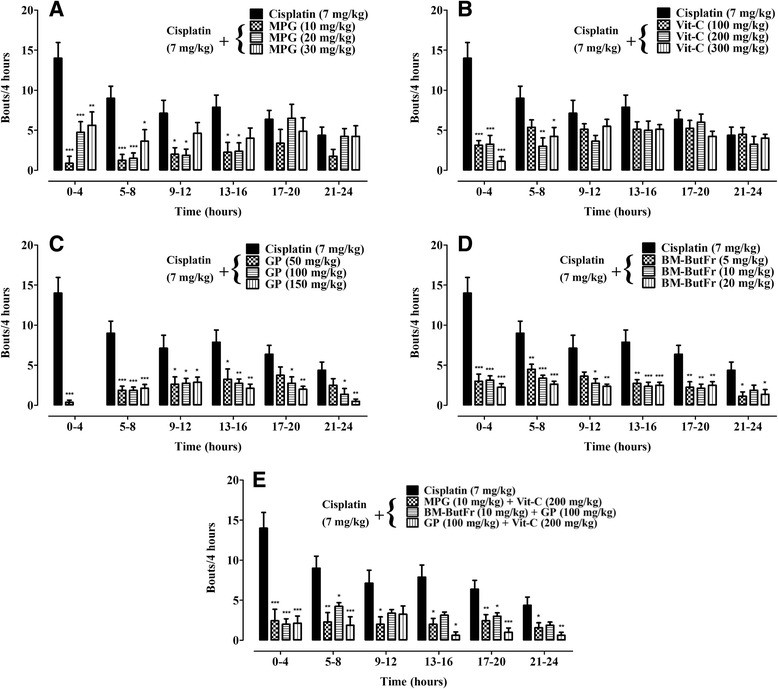
Antiemetic effect of *N*-(2-mercaptopropionyl) glycine (MPG), vitamin C (Vit-C), grape seed proanthocyanidin (GP), and *B. monnieri n*-butanolic fraction (BM-ButFr). **a** MPG (10, 20, 30 mg/kg); **b** Vit-C (100, 200, 300 mg/kg); **c** GP (50, 100, 150 mg/kg); **d** BM-ButFr (5, 10, 20 mg/kg) and **e** their combinations on cisplatin (7 mg/kg) induced vomiting during a 24 h observation period. Each bar represents mean ± S.E.M (*n* = 7–8). ^*^ *p* < 0.05, ^**^ *p* < 0.01 ^***^ *p* < 0.001 compared to cisplatin control (ANOVA followed by Tukey’s *post hoc* analysis)

As shown in Fig. 1, cisplatin generated a consistently maintained number of vomiting bouts over a 24 h period. It also induced retching; weight loss (%) and a decreased latency to first vomit (Table 1). Highly significant reductions in the number of bouts were found with MPG [*F* (3,28) = 10.62, *P* < 0.0001] (10, 20 mg/kg; 0–8 h, *P* < 0.001) (Fig. 2a), Vit-C [*F* (3,28) = 9.985, *P* = 0.0001] (100–300 mg/kg; 0–4 h, *P* < 0.001) (Fig. 2b), GP [*F* (3,28) = 50.97, *P* < 0.0001] (50–150 mg/kg; 0–8 h, *P* < 0.001) (Fig. 2c) and BM-ButFr [*F* (3,28) = 48.28, *P* < 0.0001] (5–20 mg/kg; 0–8 h and 13–16 h, *P* < 0.001) (Fig. 2d). However, the reductions were less significant (*P* < 0.01) for MPG at 30 mg/kg (0–4 h; Fig. 2a), Vit-C at 200 mg/kg (5–8 h; Fig. 2b), GP at 150 mg/kg (13–24 h; Fig. 2c) and BM-ButFr at 5–20 mg/kg (17–20 h; Fig. 2d) as compared to the cisplatin control. In the case of the combinations [*F* (3,27) = 33.55, *P* < 0.0001], highly significant inhibition (*P* < 0.001) of vomiting bouts was observed up to 4 h after cisplatin administration. The GP (100 mg/kg) + Vit-C (200 mg/kg) combination maintained marked significance at 5–8 h and during 17–20 h (*P* < 0.001) whilst the other two combinations had fluctuating inhibitory activity of low statistical significance (see Fig. 2e).

**Table 1:** Activity of *N*-(2-mercaptopropionyl) glycine (MPG), vitamin C (Vit-C), grape seed proanthocyanidin (GP), *B. monnieri n*-butanolic fraction (BM-ButFr) and their combination against cisplatin induced vomiting during a 24 h observation period

Treatment	Dose and route	Pigeons tested/vomited	Bouts	Latency (min)	Retching	Wt. loss (%)
Cisplatin	7.0 mg/kg i.v.	8/8	50 ± 3.2	70.0 ± 2.30	561 ± 56.4	16 ± 1.8
Metoclopramide	30 mg/kg i.m.	8/8	8 ± 0.8^***^	417 ± 7.00^***^	134 ± 12.5^***^	4 ± 0.6^***^
MPG	10 mg/kg i.m.	8/6	12 ± 5.8^***^	410 ± 162^*^	135 + 55.6^***^	4 ± 1.9^**^
	20 mg/kg i.m.	8/7	21 ± 4.9^**^	91.0 ± 11.5	226 + 80.9^*^	7 ± 1.2^*^
	30 mg/kg i.m.	8/7	23 ± 5.6^**^	89.0 ± 21.1	329 + 79.2	7 ± 3.2^*^
Vit-C	100 mg/kg i.m.	8/8	30 ± 3.2^**^	146 ± 16.7	384 ± 23.5^*^	7 ± 1.3^***^
	200 mg/kg i.m.	8/8	24 ± 4.6^***^	316 ± 96.2^*^	332 ± 52.4^**^	5 ± 1.1^***^
	300 mg/kg i.m.	8/8	24 ± 3.0^***^	359 ± 73.2^*^	256 ± 21.0^***^	3 ± 0.5^***^
GP	50 mg/kg i.m.	8/8	14 ± 3.5^***^	348 ± 42.5^***^	236 ± 78.9^**^	6 ± 0.8^***^
	100 mg/kg i.m.	8/8	12 ± 2.2^***^	370 ± 31.6^***^	184 ± 37.0^***^	6 ± 1.0^***^
	150 mg/kg i.m.	8/8	9.0 ± 0.9^***^	369 ± 27.8^***^	139 ± 27.2^***^	5 ± 0.5^***^
BM-ButFr	5 mg/kg i.m.	8/8	17 ± 2.6^***^	161 ± 25.0	331 ± 74.0	7 ± 0.5^**^
	10 mg/kg i.m.	8/8	15 ± 2.1^***^	145 ± 36.0	378 ± 58.0	6 ± 1.4^***^
	20 mg/kg i.m.	8/8	13 ± 1.7^***^	137 ± 25.0	253 ± 45.0^**^	5 ± 1.7^***^
Combination						
MPG + Vit-C	10 mg/kg + 200 mg/kg i.m.	7/7	12 ± 4.7^***^	421 ± 134^*^	201 ± 68.2^***^	3 ± 1.1^***^
BM-ButFr + GP	10 mg/kg + 100 mg i.m.	8/8	17 ± 0.9^***^	195 ± 24.1	196 ± 16.3^***^	5 ± 0.2^***^
GP + Vit-C	100 mg/kg + 200 mg/kg i.m.	8/8	9.0 ± 3.3^***^	309 ± 90.6	166 ± 61.3^***^	3 ± 0.9^***^

Values are expressed as mean ± S.E.M. ^*^ *P* < 0.05, ^**^ *P* < 0.01, ^***^ *P* < 0.001 compared to cisplatin control (ANOVA followed by Tukey’s *post hoc* analysis)

A 100% reduction in the frequency of cisplatin-induced vomiting bouts was observed with GP (100 and 150 mg/kg) and Vit-C (300 mg/kg). Adequate protection was also produced by MPG at 10 mg/kg (72–94%), 20 mg/kg (66–83%) and 30 mg/kg (35–60%); Vit-C at 100 mg/kg (28–78%) and 200 mg/kg (49–77%); GP at 50 mg/kg (63–97%) and BM-ButFr at 5 mg/kg (49–79%), 10 mg/kg (61–78%) and 20 mg/kg (67–84%) during the acute phase (0–12 h) of cisplatin-induced vomiting. Likewise, during the delayed phase (13–24 h), the antiemetic propensity was observed as 47–60% (10 mg/kg), 0–70% (20 mg/kg) and 3–49% (30 mg/kg) for MPG; 0–35% (100 mg/kg), 6–36% (200 mg/kg) and 8–35% (300 mg/kg) for Vit-C; 41–59% (50 mg/kg), 57–68% (100 mg/kg) and 73–88% (150 mg/kg) for GP and 65–74% (5 mg/kg), 57–70% (10 mg/kg) and 61–69% (20 mg/kg) for BM-ButFr. A highly effective percentage reduction of cisplatin-induced vomiting was afforded by the combinations of MPG (10 mg/kg) + Vit-C (200 mg/kg), BM-ButFr (10 mg/kg) + GP (100 mg/kg) and GP (100 mg/kg) + Vit-C (200 mg/kg) as 72–83%, 53–86% and 54–85% during the acute phase and 62–75%, 53–60% and 84–92% during the delayed phase, respectively (see Additional file 1).

Extremely significant reductions in the number of retching episodes was noted for MPG [*F* (3,27) = 6.639, *P* = 0.0016] (10 mg/kg, *P* < 0.001), Vit-C [*F* (3,28) = 9.658, *P* = 0.0002] (300 mg/kg, *P* < 0.001) and GP [*F* (3,28) = 12.70, *P* < 0.0001] (100, 150 mg/kg, *P* < 0.001) while less significant decrements were observed with MPG at 20 mg/kg (*P* < 0.05), Vit-C at 100 mg/kg (*P* < 0.05) and 200 mg/kg (*P* < 0.01), GP at 50 mg/kg (*P* < 0.01) and BM-ButFr [*F* (3,28) = 4.799, *P* = 0.0080] at 20 mg/kg (*P* < 0.01). Moreover, the reductions in the % weight loss were very significant with BM-ButFr [*F* (3,28) = 10.12, *P* = 0.0001] at 10, 20 mg/kg (*P* < 0.001), GP [*F* (3,28) = 18.03, *P* < 0.0001] and Vit-C [*F* (3,28) = 20.98, *P* < 0.0001] at all tested doses (*P* < 0.001). However, the decreases were less significant for MPG [*F* (3,28) = 4.938, *P* = 0.0071] at 10 mg/kg (*P* < 0.01), 20 mg/kg (*P* < 0.05) and 30 mg/kg (*P* < 0.05) and with BM-ButFr at 5 mg/kg (*P* < 0.01). Furthermore, significant increases in vomiting latency were observed with GP [*F* (3,28) = 23.93, *P* < 0.0001] at all doses (*P* < 0.001) but the increases were less significant for MPG [*F* (3,28) = 4.170, *P* = 0.0146] (10 mg/kg, *P* < 0.05) and Vit-C [*F* (3,28) = 5.048, *P* = 0.0064] (200, 300 mg/kg, *P* < 0.05) (Table 1). A tendency towards an increase in vomiting latency was observed with BM-ButFr [*F* (3,28) = 2.449, *P* = 0.084] at all doses. In the positive control group, metoclopramide (30 mg/kg) significantly alleviated (*P* < 0.001) cisplatin-induced vomiting bouts, retching and percentage weight loss, while it significantly increased (*P* < 0.001) the latency to vomiting during the entire observation period.

The number of vomiting bouts, retching [*F* (3,27) = 12.54, *P* < 0.0001] and % weight loss [*F* (3,26) = 25.72, *P* < 0.0001] were significantly reduced (*P* < 0.001) by all the combinations of selected antioxidants when compared to the cisplatin control. In addition, the vomiting latency [*F* (3,27) = 3.710, *P* = 0.0235] was significantly increased (*P* < 0.05) by the combination of MPG (10 mg/kg) + Vit-C (200 mg/kg) (Table 1).

The global reduction in the number of emetic bouts for the selected antioxidants and their combinations decreased in the following respective rank orders: GP > BM-ButFr > MPG > Vit-C and GP + Vit-C > MPG + Vit-C > BM-ButFr + GP.

### In vitro antioxidant activity of N-(2-mercaptopropionyl) glycine (MPG), vitamin C (Vit-C), grape seed proanthocyanidin (GP) and B. monnieri n-butanolic fraction (BM-ButFr)

**Fig. 3:**
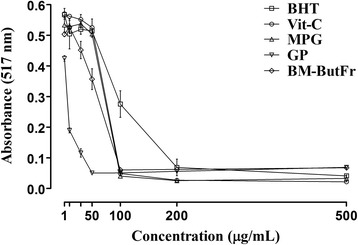
DPPH free radical scavenging assay in vitro showing absorbance of *N*-(2-mercaptopropionyl) glycine (MPG), vitamin C (Vit-C), grape seed proanthocyanidin (GP), *B. monnieri n*-butanolic fraction (BM-ButFr) or standard (butylated hydroxytoluene: BHT) against their respective concentrations. Data are presented as mean ± SD of three separate experiments

The maximum inhibition of DPPH free radicals by BHT (standard) was 93.82% at 500 μg/mL while those of MPG, Vit-C, GP and BM-ButFr were 96.15% at 200 μg/mL, 96.71% at 500 μg/mL, 92.42% at 50 μg/mL and 90.94% at 100 μg/mL respectively as shown in Table 2. MPG, Vit-C, GP and BM-ButFr or standard (BHT) exhibited concentration dependent declines in spectral absorbance (Fig. 3). The EC_50_, antiradical power and stoichiometry of MPG, Vit-C, GP, BM-ButFr and BHT are shown in Table 2 and the antioxidant activity, established by EC_50_ values was decreased in the following rank order: GP > BM-ButFr > MPG > Vit-C > BHT.

**Table 2:** Percent of DPPH free radical scavenging activity and antioxidant strength of A-(2-mercaptopropionyl) glycine (MPG), vitamin C (Vit- C), grape seed proanthocyanidin (GP), *B. monnieri* n-butanolic fraction (BM-ButFr) or standard butylated hydroxytoluene (BHT) against their respective concentrations

Concentration (pg/mL)	Percent inhibition (%)				
	BHT	MPG	Vit-C	GP	BM-ButFr
1	15.0 ± 0.7	20.0 ± 2.1	14.6 ± 1.5	36.4 ± 0.8	24.5 ± 0.7
10	24.4 ± 4.2	20.8 ± 1.8	15.7 ± 0.6	71.6 ± 0.7	20.9 ± 0.8
30	22.1 ± 1.4	19.4 ± 1.2	17.3 ± 1.5	82.6 ± 1.2	32.2 ± 2.4
50	22.8 ± 0.5	24.4 ± 1.0	21.2 ± 2.4	92.4 ± 0.1	46.5 ± 2.9
100	58.6 ± 3.8	94.0 ± 0.01	92.7 ± 0.7	92.4 ± 0.2	90.9 ± 0.5
200	89.7 ± 2.4	96.2 ± 0.2	96.0 ± 0.3	91.6 ± 0.3	90.7 ± 0.3
500	93.8 ± 0.1	96.0 ± 0.7	96.7 ± 0.2	89.6 ± 0.2	89.9 ± 0.4
Antioxidant strength					
EC50 (pg/mL)	98.17 ± 3.842	67.66 ± 3.095	69.42 ± 3.027	6.498 ± 0.630	55.61 ± 1.137
Antiradical power	0.0102 ± 0.0004	0.0148 ± 0.0007	0.0143 ± 0.0005	0.1567 ± 0.0145	0.0159 ± 0.0010
Stoichiometry	196.3 ± 7.685	135.3 ± 6.191	138.8 ± 6.053	13.00 ± 1.261	111.2 ± 2.274

Values are expressed as mean ± S.E.M from three separate experiments

### Correlations between in vitro antioxidant and in vivo antiemetic activities

**Fig. 4:**
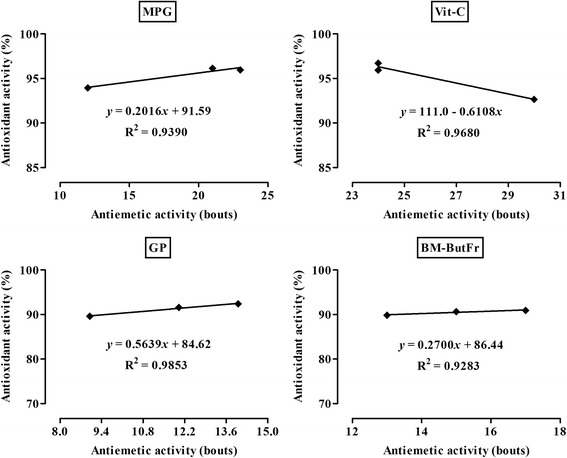
Linear correlation showing the involvement of in vitro antioxidant activities of *N*-(2-mercaptopropionyl) glycine (MPG), vitamin C (Vit-C), grape seed proanthocyanidin (GP) and *B. monnieri n*-butanolic fraction (BM-ButFr) in the reduction of cisplatin induced vomiting bouts in pigeons

The antiemetic activities in vivo were correlated with the free radical scavenging capacities of the natural and synthetic antioxidants in vitro. Figure 4 shows the high degree of Pearson correlation between the number of vomiting bouts and the % of maximum free radical scavenging capacity of the selected antioxidants. The results manifested positive correlation coefficients for MPG (*r* = 0.9690), GP (*r* = 0.9926) and BM-ButFr (*r* = 0.9635). However, a negative correlation coefficient between the number of bouts and antioxidant activity was observed with Vit-C (*r* = − 0.9838). The coefficients of determination (R^2^: a statistical measure of how well the regression lines represent the data), disclosed an association between antioxidant activity and antiemetic assay outcome. This deduction was substantiated by the values obtained for MPG (*R* ^2^ = 0.9390), Vit-C (*R* ^2^ = 0.9680), GP (*R* ^2^ = 0.9853) and BM-ButFr (*R* ^2^ = 0.9283) (Fig. 4).

## Discussion

In this study, different antioxidants of natural and synthetic origin were evaluated for their antiemetic activity against cisplatin-induced retching and vomiting in pigeons. In emetogenesis studies, different animal models including monkeys [45], pigs [46], ferrets [47], dogs [48], cats [49], house musk shrews [50] and rats [51] have been utilized for evaluating antiemetic compound activity. However, these models have some limitations in terms of cost, ease of handling, absence of a vomiting center, and inability to vomit. We have chosen the pigeon emesis model due to the fact that it expresses readily quantifiable vomiting response parameters as reported in previous studies [3, 52]. The pigeon responds to a number of different emetic stimuli, including cardiac glycosides [53], reserpine [54], sigma receptor ligands [55], 5-HT_3_ receptor agonists [56] and chemotherapeutic drugs [57]. The pigeon model can also be used to assay the antiemetic activity of several classes of drugs for example NK_1_ receptor antagonists [58] and glucocorticoids [59].

Vomiting induced by cisplatin is biphasic with an acute phase lasting for 24 h and a delayed phase extending to several days [2]. In pigeons, there is no mechanistically distinct acute or delayed phase of chemotherapy-induced vomiting, although earlier studies have monitored emesis for up to 72 h [59]. In our investigation, we observed the animals for 24 h in order to comply with the ethical use of animals. The dose of cisplatin for induction of emesis varies with the animal model [45, 60, 61]. We utilized cisplatin at a dose of 7.0 mg/kg for pigeons [39] and observed a robust elevation in the number of vomiting bouts, retching and % weight loss after 24 h.

Cancer chemotherapy is associated with generation of reactive oxygen species [62] and oxidative stress has been implicated in the emesis caused not only by cisplatin but other chemotherapeutic drugs as well [63]. Numerous studies have shown that the active metabolite of cisplatin i.e. cis-diaqodiammineplatinum generates free radicals that release serotonin from enterochromafin cells which then stimulate 5-HT_3_ receptors on vagal afferents and initiate the emetic reflex within the brain stem [64, 65]. Since the emetogenic effect of cisplatin is associated with the generation of reactive oxygen species (ROS), administration of antioxidants could detoxify ROS and thereby prevent cisplatin-induced emesis. Accordingly, antioxidants and free radical scavengers have been shown to increase the therapeutic efficacy of chemotherapy by improving tolerance and reducing their dose limiting toxicities [66]. In the present study, we have examined well established synthetic and natural antioxidants, including MPG, Vit-C, GP and BM-ButFr. Moreover, the doses chosen for these antioxidants were tolerable and benign based on their toxicity profiles [67–70].

All the selected antioxidants at the doses tested significantly decreased the number of vomiting bouts, retching and % weight loss whilst at the same time, increasing the latency to vomiting. The most intense antiemetic effect was observed with GP followed by BM-ButFr, MPG then Vit-C and our study is the first to report the antiemetic activity of GP in the pigeon vomit model. GP at doses of 100 and 150 mg/kg produced a complete reversal of emesis as seen by a 100% reduction in the frequency of cisplatin-induced vomiting bouts. Previously, GP at 10 mg/kg has been shown to produce a significant reduction of cisplatin-induced pica behavior in rats and this is exemplified by a decreased kaolin intake [30], which is regarded as being analogous to emesis [51]. Proanthocyanidin, at 200 mg/kg, ameliorated the cisplatin-induced decrease in the activities of antioxidant enzymes, GSH, total protein and albumin [31] while at 250 mg/kg, it alleviated cisplatin-induced hepatotoxicity in rabbits by reducing ROS generation and strengthening endogenous antioxidant systems [32]. It is noteworthy that in rodents, investigations employing a similar dose of grape seed proanthocyanidin as that used in our study, reported beneficial effects which have been attributed to an ability to support the antioxidant defense system [41, 71].

Different plants have been screened against cisplatin-induced emesis in a variety of animal models. These include *Zingiber officinale* Rosc. (Zingiberaceae) [48], *Scutellaria baicalensis* Georgi (Lamiaceae) [72] and American ginseng berry (*Panax quinquefolius* L.) (Araliaceae) [73]. In these studies, the antiemetic effect has been attributed to the free radical scavenging property and an antiserotonergic action of the different active constituents. In the current investigation, the *n*-butanolic fraction of *B. monnieri* (BM-ButFr) exhibited a dose dependant antiemetic activity and this accords with our previous report in which BM-ButFr significantly reduced cisplatin-induced emetogenesis [39]. The superior antiemetic effect of BM-ButFr compared to the synthetic antioxidants seen here, may be ascribed to the presence of bacoside-A components, which along with bacopaside I, constitute more than 96% w/w of the total saponins present in the extract [74]. However, the antiemetic activity of *B. monnieri* may also be mediated through other mechanisms in addition to its antioxidant activity [75] because *B. monnieri* has both anti-dopaminergic and anti-serotonergic properties [76]. In relation to this, serotonin [77] and dopamine [78] both play an important role in the induction of vomiting at the level of the area postrema. However, serotonin has been shown to differentially mediate the early emetic phase following cisplatin treatment [77]. In line with this, our previous study showed that *B. monnieri* not only attenuated the cisplatin-induced dopamine upsurge in the area postrema and brain stem but it also diminished the intestinal serotonin concentration in the pigeons [39].

MPG is a well-known synthetic aminothiol antioxidant that has been studied widely especially for its cardioprotective properties [78, 79]. In the present pigeon emetogenesis model, it produced a significant decline in frequencies of cisplatin-induced retching and vomiting and this is consistent with previous reports in such species as dogs [25], rats [80] and *Suncus murinus* [26]. It is clear that MPG has some proficiency in scavenging generated free radicals and that it exerts its beneficial effects by protecting against oxidative stress [78]. What is more, our study revealed that MPG is effective when given in lower doses since in a higher dose MPG itself causes emesis [25].

Vitamin C is a versatile water soluble antioxidant that is widely used in complementary oncology [81]. In our study, it significantly attenuated cisplatin-induced emetic episodes and this is in accordance with earlier accounts describing diminished cisplatin emesis in dogs and it also inhibited kaolin consumption in cisplatin treated rats [25, 80]. Moreover, at 300 mg/kg, Vit-C produced a 100% reduction of emesis. In relation to such findings, vitamin C has efficacy in reducing cisplatin oxidative stress by improving antioxidant levels, repairing DNA damage and inhibiting lipid peroxidation [82].

We have screened combinations of GP + Vit-C, MPG + Vit-C and BM-ButFr + GP against cisplatin-induced retching and vomiting. Our results showed that the GP + Vit-C combination yielded an equivalent inhibition of emetic episodes to MPG + Vit-C and BM-ButFr + GP. Additionally, the combinations tended to exert comparable, or in some instances marginally improved antiemetic effects than either agent given alone. In this respect, it has been reported that combination of vitamin E plus vitamin C affords enhanced protection against emetic episodes in dogs [25]. Similarly, a combination of vitamins C and E provides superior antiemetic activity than either of the antioxidants alone in cisplatin-induced pica behavior in rats [80]. No single antiemetic is completely effective at blocking emesis in either phase, but when administered together, the antiemetic efficacy of the combination is often greater than that of each agent given individually [77].

The inherent antioxidant potential of MPG, Vit-C, GP and BM-ButFr was evaluated by the DPPH free radical scavenging assay as this method is considered as one of the standard colorimetric methods for the evaluation of antioxidant properties of natural and pure compounds [83]. The antioxidant activity of the tested compounds was quantified in terms of EC_50_, antiradical power and stoichiometry. Agents with a low EC_50_/stoichiometry value plus high antiradical power indicated strong antioxidant activity [83, 84]. Consequently, a distinct antioxidant capability was observed for GP which yielded EC_50_, antiradical power and stoichiometry values of 6.498, 0.1567 and 13.00 respectively, and this was followed in rank order by BM-ButFr (55.61, 0.0159, 111.2), MPG (67.66, 0.0148, 135.3) and Vit-C (69.42, 0.0143, 138.8). These results indicated that as compared to the standard (BHT), all the selected antioxidants possessed strong free radical scavenging capacities, as seen in previous studies [43, 85].

The rank order of antioxidant activity in vitro correlated well with in vivo antiemetic activity, GP having strong antioxidant and antiemetic proclivity among the selected agents. Several studies have reported that antioxidant properties of various plants or synthetic compounds significantly contribute to their antiemetic activities [25, 86]. In the current study, the high degree of correlation between the antioxidant and antiemetic activities as evidenced by their coefficients of determination which implied that amelioration of cisplatin emetogenesis could at least be partially ascribed to the potent free radical scavenging capacities of GP, MPG, BM-ButFr and Vit-C. A substantial body of evidence suggests that oxidative stress is one of the triggering mechanisms in the mediation of vomiting induced by chemotherapeutic agents such as cisplatin [87, 88]. Cisplatin induces lipid peroxidation in the brain, liver and small intestine and releases serotonin by generating free radicals. Antioxidants scavenge the generated free radicals and protect the enterochromafin cells from oxidative injury thereby suppressing the release of serotonin in the emetogenic pathway [26]. Free radical mediated reactions are responsible for a wide range of chemotherapy-induced side effects and antioxidants are able to protect non-malignant cells and organs against some of the damaging effects of cytostatic agents [63]. Dietary supplementation with synthetic and herbal antioxidants ameliorates chemotherapy-induced oxidative stress and diminishes the development of their side effects as well as improving the overall response to therapy [89]. Our study therefore endorses the notion that free radical scavengers may be a beneficial class of prophylactic drugs against cancer chemotherapeutic drug induced emesis. However, further studies are warranted to investigate if there is direct evidence linking reactive oxygen species with oxidative/redox stress injury by assay of oxidized lipid or protein markers after cisplatin treatment. Ultimately, neurochemical analysis should be performed to further correlate the antiemetic effect of these antioxidants with behavioral parameters in this model of emesis.

## Conclusions

Cisplatin treatment was associated with intense vomiting as exemplified by a significant increase in the number of emetic bouts, retching, % weight loss and a simultaneous decrease in the emetic latency in the pigeon model. Pretreatment with MPG, Vit-C, GP, and BM-ButFr or their combinations significantly attenuated cisplatin-induced elevation of vomiting episodes. The selected agents all possessed potent free radical scavenging capability and consequent antioxidant potential as evaluated via the DPPH free radical scavenging assay. Although all agents exhibited efficacy, GP was conspicuous with respect to antiemetic and antioxidant potential. Evaluation of correlation coefficients disclosed close linear relationships between antioxidant and antiemetic propensity emphasizing the involvement of antioxidant activity in the reduction of cisplatin-induced retching and vomiting episodes for all the agents tested in the study.

## Additional file

Additional file 1: Effect of synthetic and natural antioxidants on cisplatin-induced vomiting bouts. Bouts per hour graph for: *N*-(2-mercaptopropionyl) glycine (MPG) at 10 mg/kg (**Figure S1**), 20 mg/kg (**Figure S2**) and 30 mg/kg (**Figure S3**); Vitamin C (Vit-C) at 100 mg/kg (**Figure S4**), 200 mg/kg (**Figure S5**) and 300 mg/kg (**Figure S6**); Grape seed proanthocyanidin (GP) at 50 mg/kg (**Figure S7**), 100 mg/kg (**Figure S8**) and 150 mg/kg (**Figure S9**); *Bacopa monnieri n*-butanolic fraction (BM-ButFr) at 5 mg/kg (**Figure S10**), 10 mg/kg (**Figure S11**) and 20 mg/kg (**Figure S12**). (DOC 3011 kb)

## Abbreviations

5-HT_3_: Serotonin 5-HT_3_ receptor
BHT: Butylated hydroxytoluene
BM-ButFr: *Bacopa monnieri n*-butanolic fraction
DPPH: 2,2-diphenyl-1-picrylhydrazyl
FRSA: DPPH free radical scavenging assay
GP: Grape seed proanthocyanidin
GSH: Glutathione
MPG: *N*-(2-mercaptopropionyl) glycine
ROS: Reactive oxygen species
Vit-C: Vitamin C
