# Erratum to: Synthetic and natural antioxidants attenuate cisplatin-induced vomiting

**DOI:** 10.1186/s40360-017-0117-x

**Published:** 2017-02-01

**Authors:** Javaid Alam, Fazal Subhan, Ihsan Ullah, Muhammad Shahid, Gowhar Ali, Robert D. E. Sewell

**Affiliations:** 1 Department of Pharmacy, University of Peshawar, Peshawar, 25120 Khyber Pakhtunkhwa Pakistan; 2 Department of Pharmacy, University of Swabi, Swabi, Pakistan; 3 Cardiff School of Pharmacy and Pharmaceutical Sciences, Cardiff University, Cardiff, CF103NB UK

## Erratum

After publication of the original article [1], it was brought to our attention that the unit for concentration in the first column of Table 2 should read μg/mL. In addition, the 9^th^ row in Table 2 should read EC_50_ (μg/mL).

**Table 2:** Percent of DPPH free radical scavenging activity and antioxidant strength of N-(2-mercaptopropionyl) glycine (MPG), vitamin C (Vit- C), grape seed proanthocyanidin (GP), *B. monnieri* n-butanolic fraction (BM-ButFr) or standard butylated hydroxytoluene (BHT) against their respective concentrations

Concentration (μg/mL)	Percent inhibition (%)				
	BHT	MPG	Vit-C	GP	BM-ButFr
1	15.0 ± 0.7	20.0 ± 2.1	14.6 ± 1.5	36.4 ± 0.8	24.5 ± 0.7
10	24.4 ± 4.2	20.8 ± 1.8	15.7 ± 0.6	71.6 ± 0.7	20.9 ± 0.8
30	22.1 ± 1.4	19.4 ± 1.2	17.3 ± 1.5	82.6 ± 1.2	32.2 ± 2.4
50	22.8 ± 0.5	24.4 ± 1.0	21.2 ± 2.4	92.4 ± 0.1	46.5 ± 2.9
100	58.6 ± 3.8	94.0 ± 0.01	92.7 ± 0.7	92.4 ± 0.2	90.9 ± 0.5
200	89.7 ± 2.4	96.2 ± 0.2	96.0 ± 0.3	91.6 ± 0.3	90.7 ± 0.3
500	93.8 ± 0.1	96.0 ± 0.7	96.7 ± 0.2	89.6 ± 0.2	89.9 ± 0.4
Antioxidant strength					
EC_50_ (μg/mL)	98.17 ± 3.842	67.66 ± 3.095	69.42 ± 3.027	6.498 ± 0.630	55.61 ± 1.137
Antiradical power	0.0102 ± 0.0004	0.0148 ± 0.0007	0.0143 ± 0.0005	0.1567 ± 0.0145	0.0159 ± 0.0010
Stoichiometry	196.3 ± 7.685	135.3 ± 6.191	138.8 ± 6.053	13.00 ± 1.261	111.2 ± 2.274

Values are expressed as mean ± S.E.M from three separate experiments

The original article was corrected. The corrections have been published in this erratum for quick reference. We apologise for any confusion this may have caused.
