# Supplementary material for: Synthetic and natural antioxidants attenuate cisplatin-induced vomiting
Source: BMC Pharmacol Toxicol. 2017 Jan 13;18:4. doi: 10.1186/s40360-016-0110-9 (PMC5234122; doi:10.1186/s40360-016-0110-9)
Supplement: Additional file 1: — Effect of synthetic and natural antioxidants on cisplatin-induced vomiting bouts. Bouts per hour graph for: N-(2-mercaptopropionyl) glycine (MPG) at 10 mg/kg (Figure S1), 20 mg/kg (Figure S2) and 30 mg/kg (Figure S3); Vitamin C (Vit-C) at 100 mg/kg (Figure S4), 200 mg/kg (Figure S5) and 300 mg/kg (Figure S6); Grape seed proanthocyanidin (GP) at 50 mg/kg (Figure S7), 100 mg/kg (Figure S8) and 150 mg/kg (Figure S9); Bacopa monnieri n-butanolic fraction (BM-ButFr) at 5 mg/kg (Figure S10), 10 mg/kg (Figure S11) and 20 mg/kg (Figure S12). (DOC 3011 kb) [file 40360_2016_110_MOESM1_ESM.doc]

**Synthetic and natural antioxidants attenuate cisplatin-induced vomiting**

Javaid Alam^1^, Fazal Subhan^1^, Ihsan Ullah^2^, Muhammad Shahid^1^, Gowhar Ali^1^, Robert D. E. Sewell^3^

**Affiliations**

^1^Department of Pharmacy, University of Peshawar, Peshawar, Pakistan

^2^Department of Pharmacy, University of Swabi, Swabi, Pakistan

^3^Cardiff School of Pharmacy and Pharmaceutical Sciences, Cardiff University, Cardiff CF103NB, UK

**Email addresses**

Javaid Alam: javaid_pharmacist@hotmail.com

Fazal Subhan: fazal_subhan@upesh.edu.pk

Ihsan Ullah: ihsanmkd@gmail.com

Muhammad Shahid: shahidsalim_2002@hotmail.com

Gowhar Ali: gohar.pharmacist@gmail.com

Robert D. E. Sewell: Sewell@cardiff.ac.uk

**Corresponding author**

Professor Fazal Subhan

Department of Pharmacy

University of Peshawar

Peshawar 25120

Khyber Pakhtunkhwa, Pakistan

Email: fazal_subhan@upesh.edu.pk

Cell: +92-3018805966

Phone: +92-919216750

**Effect of synthetic and natural antioxidants on cisplatin-induced vomiting bouts**

**Effect of *N*-(2-mercaptopropionyl) glycine (MPG) on cisplatin-induced vomiting**

**
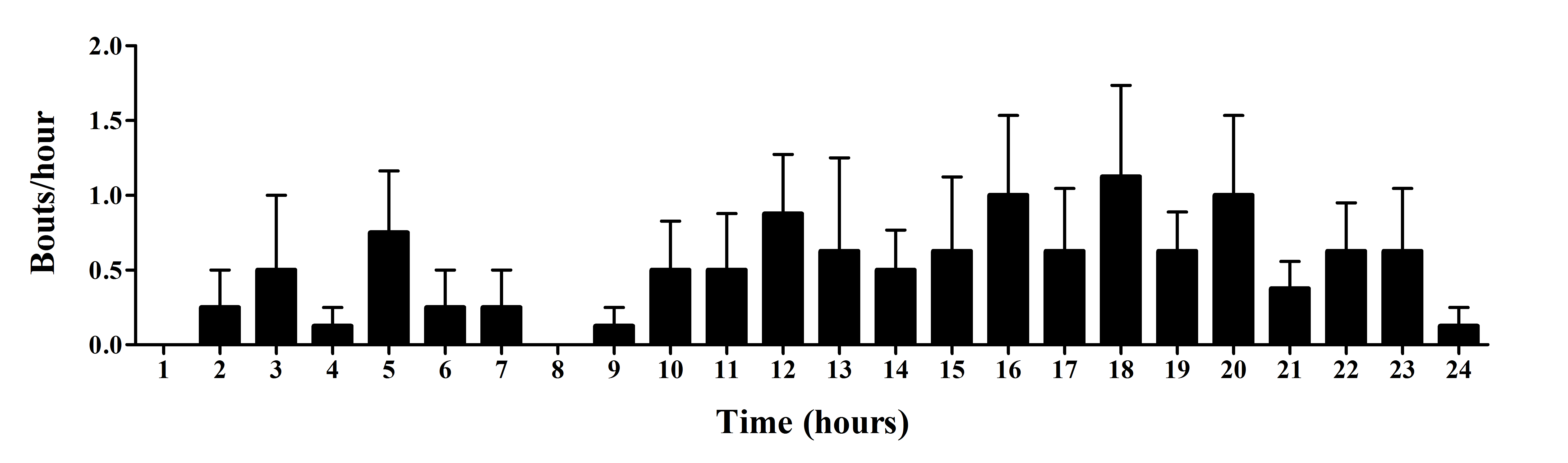
**

**Figure S1:** Bouts per hour graph for MPG at 10 mg/kg.


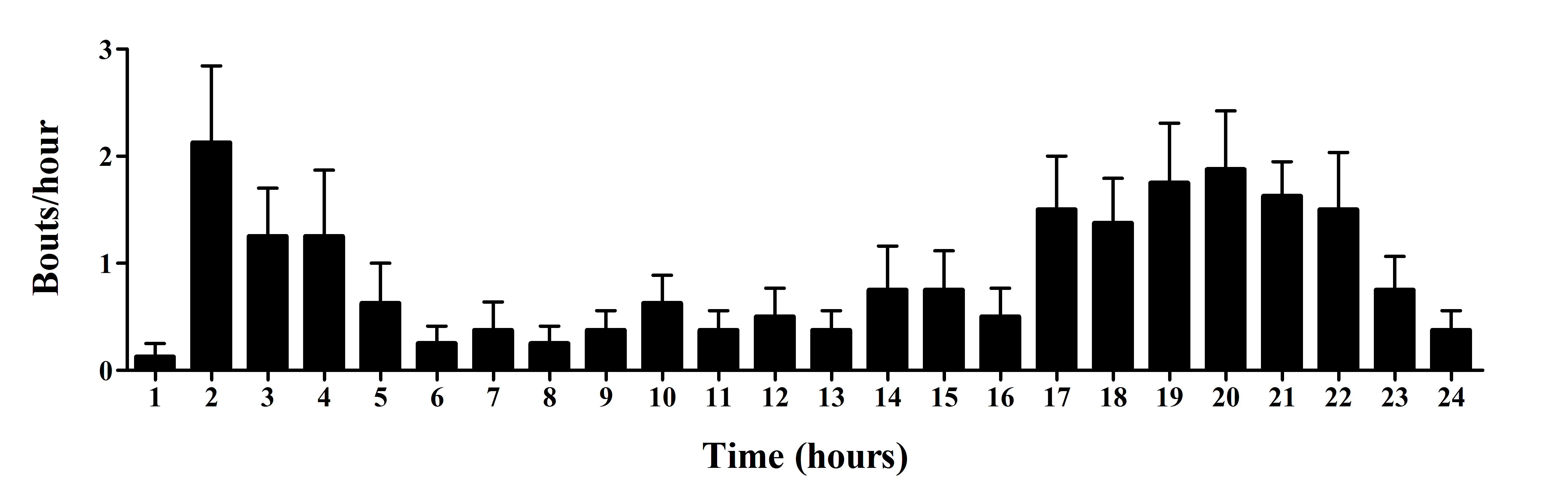


**Figure S2:** Bouts per hour graph for MPG at 20 mg/kg.


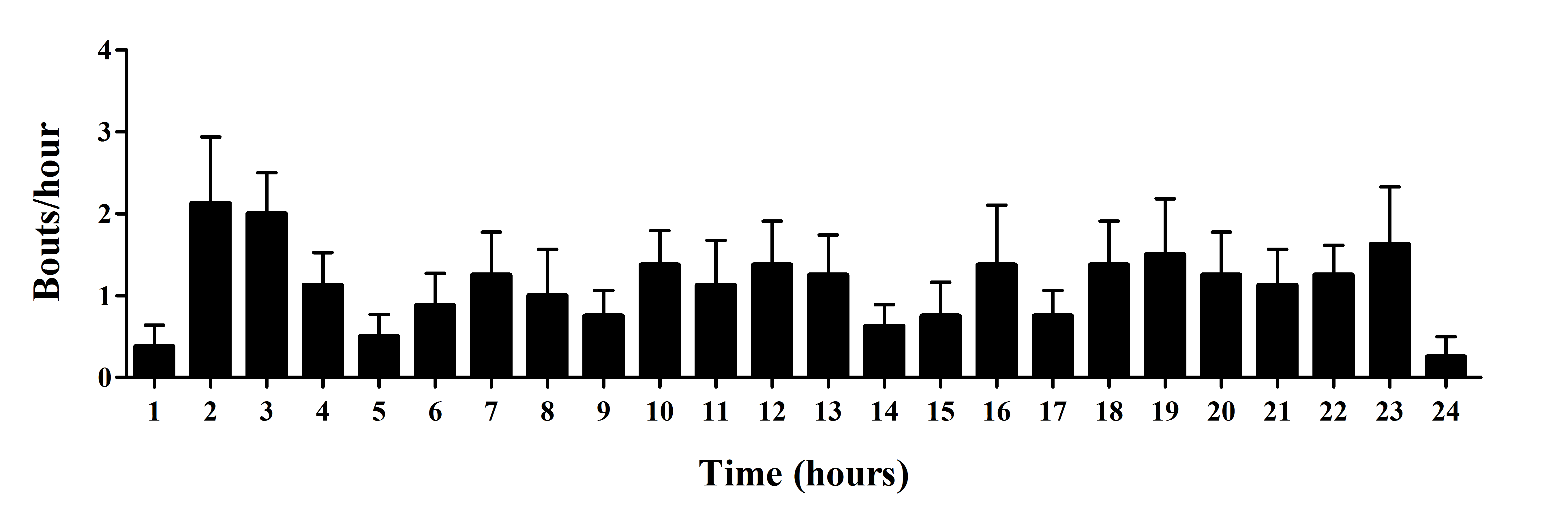


**Figure S3:** Bouts per hour graph for MPG at 30 mg/kg.

**Effect of vitamin C (Vit-C) on cisplatin-induced vomiting**

**
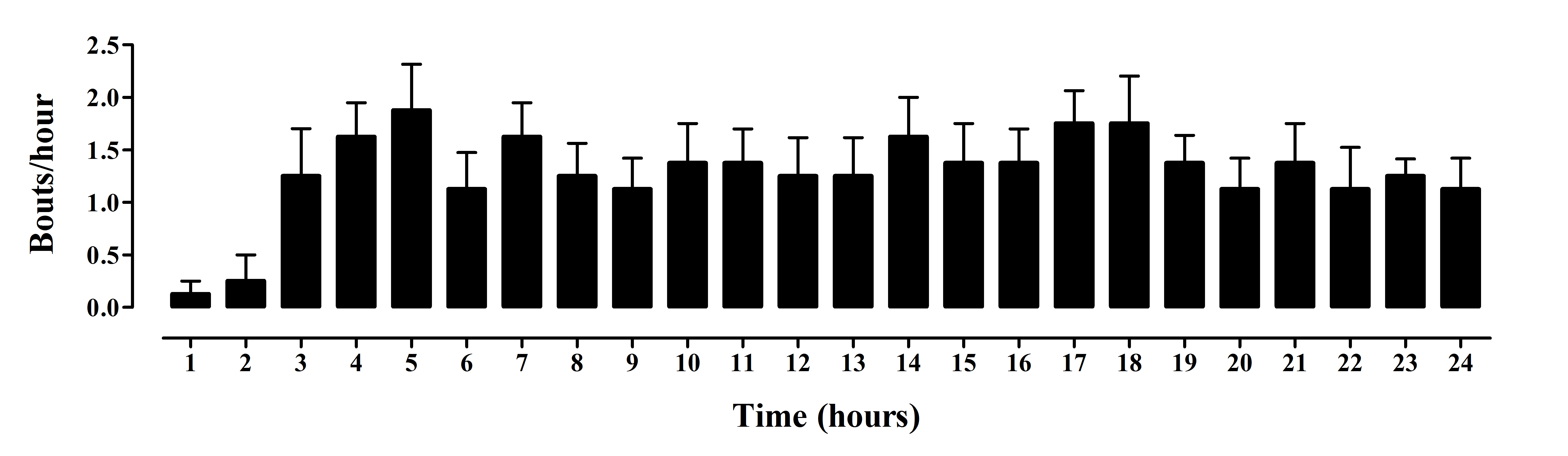
**

**Figure S4:** Bouts per hour graph for Vit-C at 100 mg/kg.


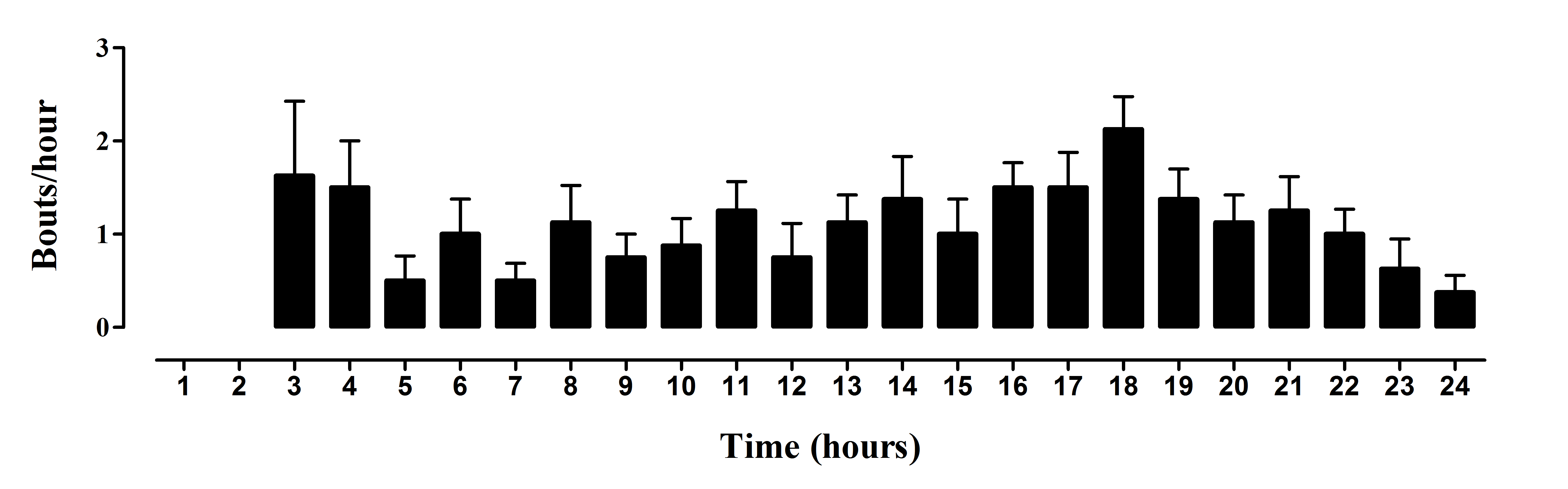


**Figure S5:** Bouts per hour graph for Vit-C at 200 mg/kg.


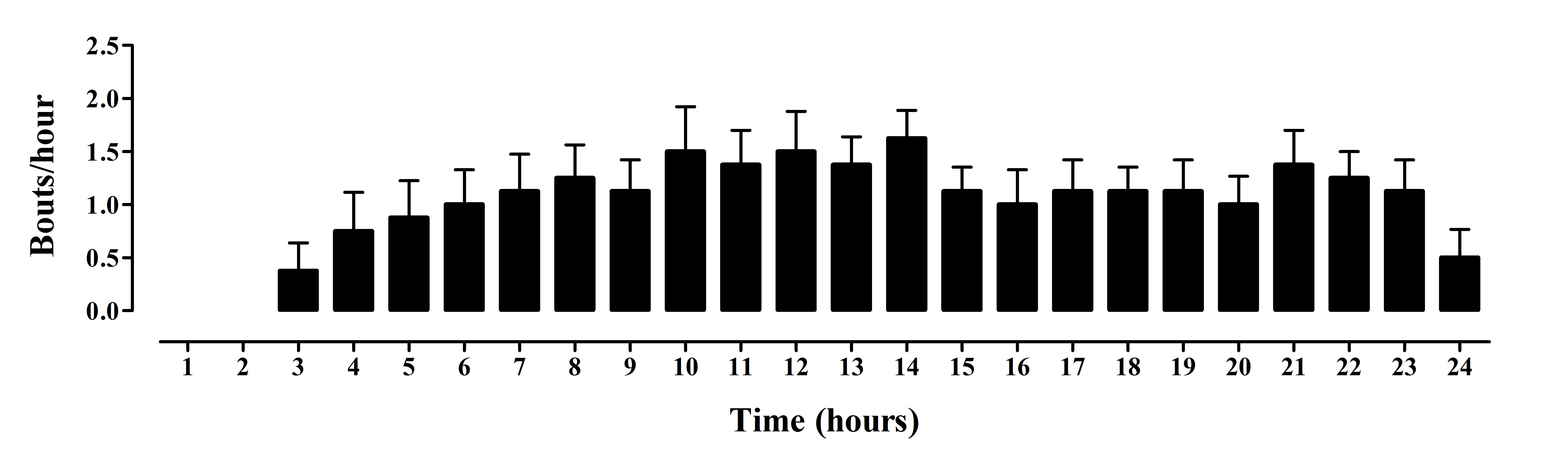


**Figure S6:** Bouts per hour graph for Vit-C at 300 mg/kg.

**Effect of grape-seed proanthocyanidin (GP) on cisplatin-induced vomiting**

**
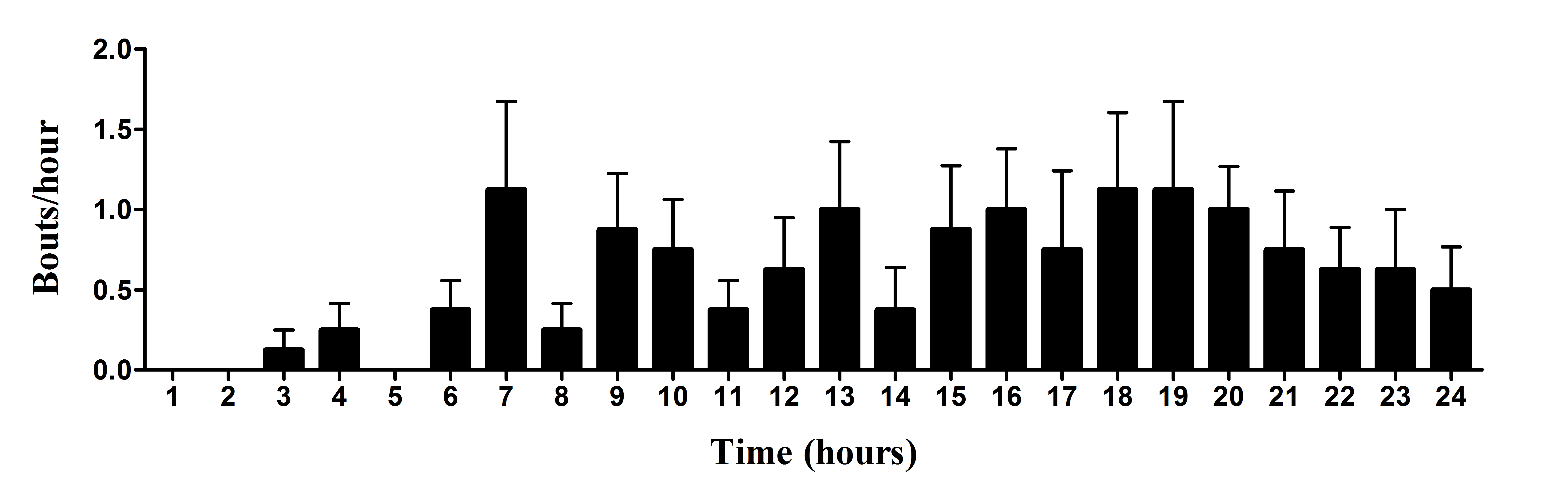
**

**Figure S7:** Bouts per hour graph for GP at 50 mg/kg.


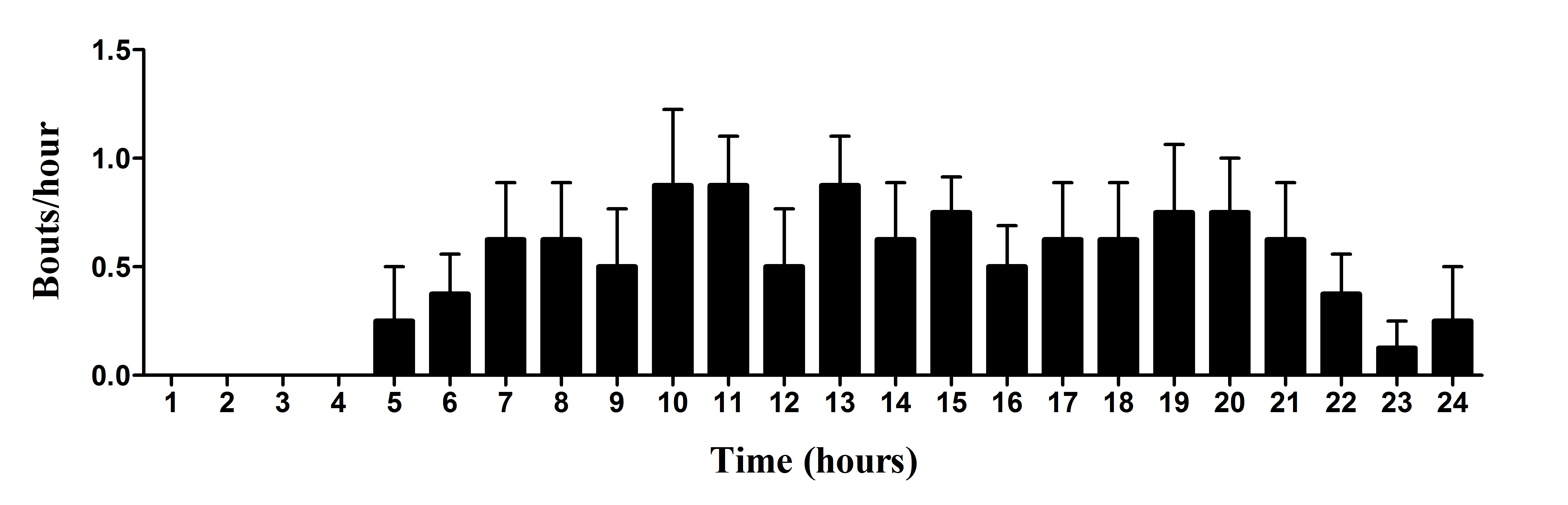


**Figure S8:** Bouts per hour graph for GP at 100 mg/kg.


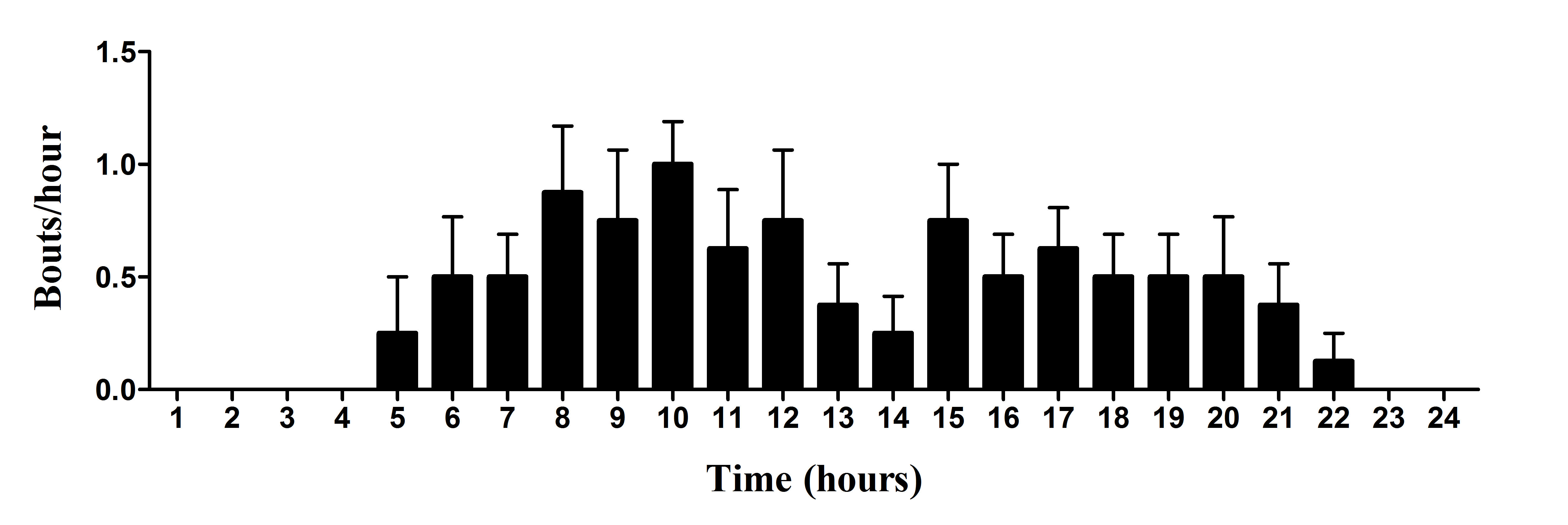


**Figure S9:** Bouts per hour graph for GP at 150 mg/kg.

**Effect of *Bacopa monnieri* butanolic fraction (BM-ButFr) on cisplatin-induced vomiting**

**
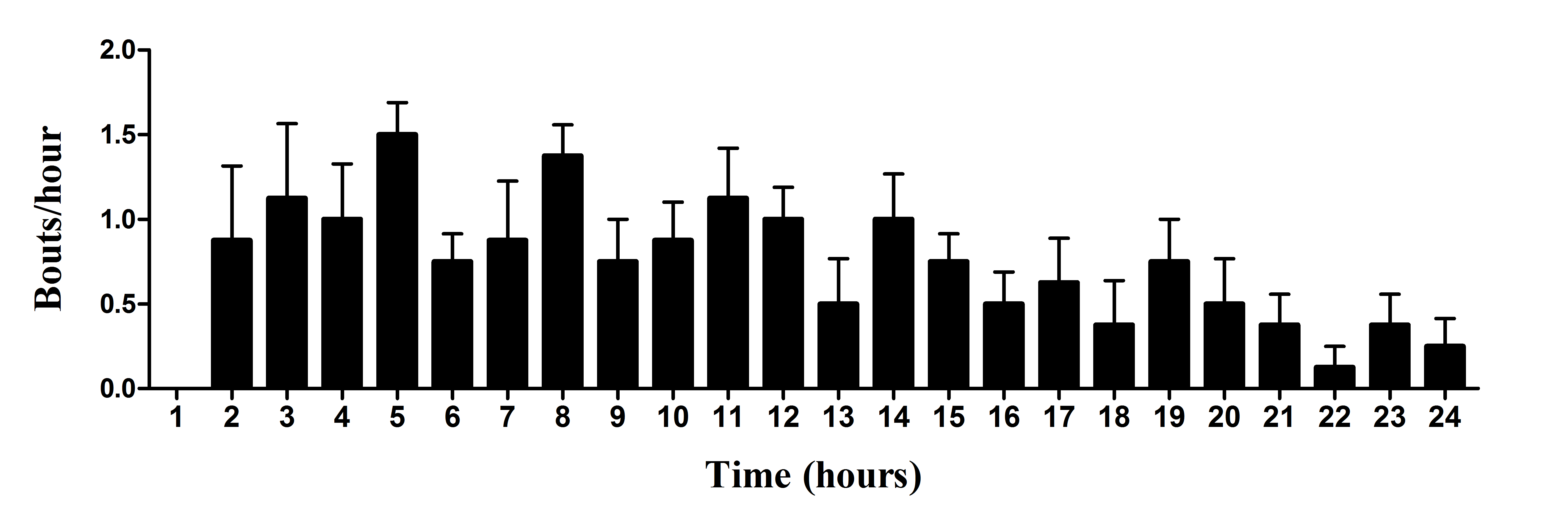
**

**Figure S10:** Bouts per hour graph for BM-ButFr at 5 mg/kg.

**
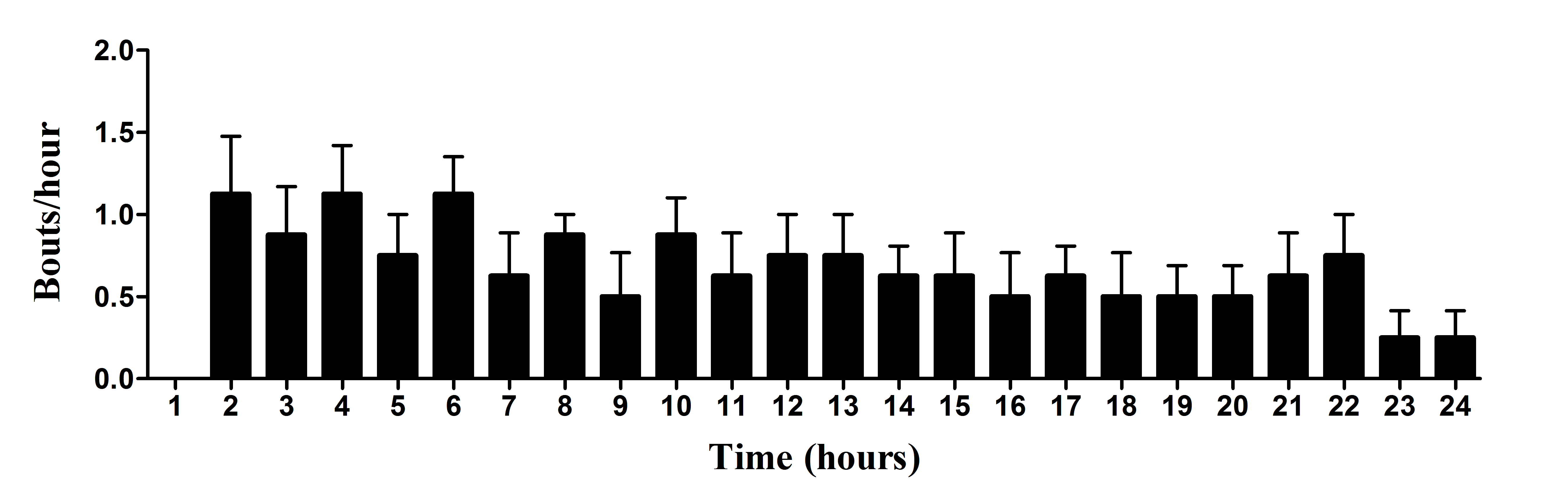
**

**Figure S11:** Bouts per hour graph for BM-ButFr at 10 mg/kg.

**
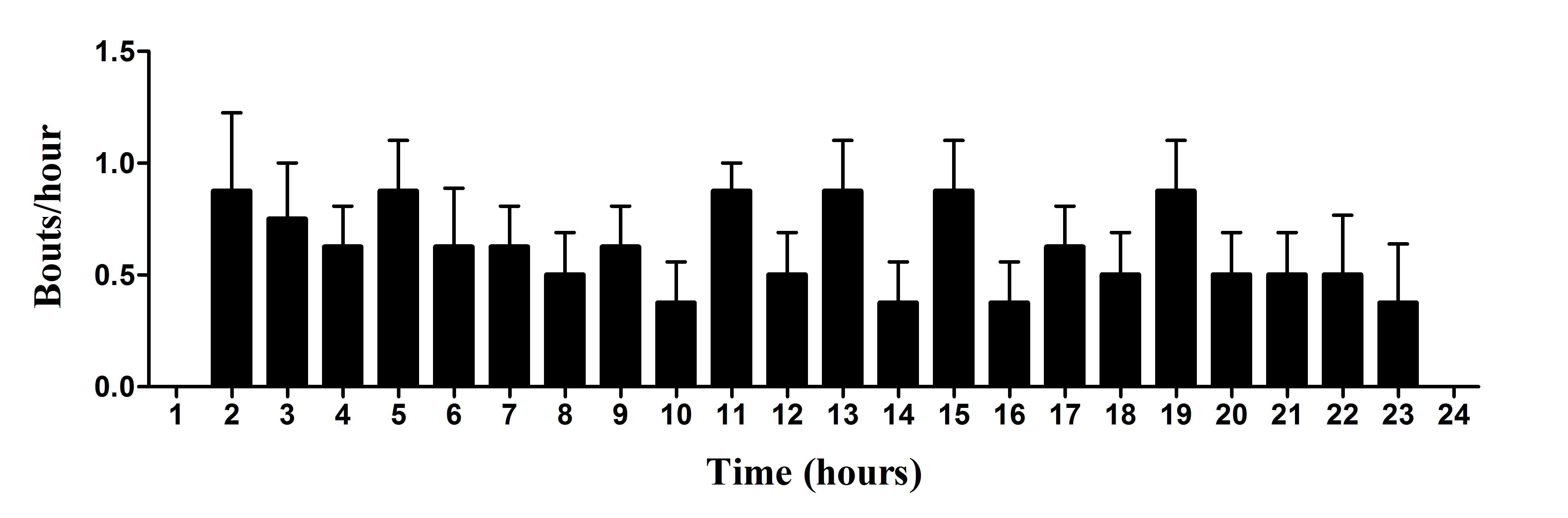
**

**Figure S12:** Bouts per hour graph for BM-ButFr at 20 mg/kg.
